# Diabetic Striatopathy: Case Report and Possible New Actors

**DOI:** 10.1155/2022/4176419

**Published:** 2022-12-19

**Authors:** Chiara Mozzini, Raffaele Ghirardi, Mauro Pagani

**Affiliations:** ^1^Department of Medicine, ASST Mantova, C. Poma Hospital Strada Lago Paiolo, Mantova 104600, Italy; ^2^Department of Medicine, ASST Mantova, Presidio di Pieve, Via Bugatte, Borgo Mantovano, Mantova 146036, Italy

## Abstract

Diabetic striatopathy is a very rare neurological complication of diabetes. We report the case of an 86-year-old woman with poorly controlled type 2 diabetes admitted to the internal medicine ward for sudden onset of altered sensorium and severe bilateral choreiform and ballistic movements. The precise pathophysiology of this condition is not well understood. Our communication aims to remind clinicians to consider the possibility of diabetic striatopathy when poor-controlled diabetic patients have sudden-onset choreiform and ballistic movements. Moreover, this case suggests the possibility that oxidative and endoplasmic reticulum stress may be involved in this process.

## 1. Introduction

Diabetic striatopathy (DS) is a very rare neurological complication of diabetes. The classic clinical manifestation of DS includes the triad of acute or subacute hemichorea-hemiballismus, hyperglycemic state, and unique reversible abnormalities limited to the striatum on neuroimaging [1]. It was first described in 1960 by Bedwell [2].

This case report aims to remind clinicians to consider DS when poor-controlled diabetic patients have sudden-onset choreiform and ballistic movements. Moreover, authors suggest the possibility that oxidative and endoplasmic reticulum stress may be involved in the pathogenesis of DS.

## 2. Case Presentation

### 2.1. Clinical Presentation

We report the case of an 86-year-old woman with poorly controlled type 2 diabetes admitted to the internal medicine ward for the sudden onset of altered sensorium and severe bilateral choreiform and ballistic movements. She had previously been admitted to the emergency department.

The patient had a history of hypertension, chronic coronary artery disease, and chronic atrial fibrillation. The diabetes was home-treated with repaglinide.

### 2.2. Laboratory Results

Laboratory results showed leukocytosis elevated *C*-reactive protein, hyperglycemia, and a positive urine test for infection. No faecal occult bloods was not detected nor were there any visible gastroenterological losses but iron, folic acid, and B12 vitamin deficiencies were detected (the main laboratory tests at admission and at discharge are depicted in Table 1). Ferritin values were in the normal range, likely due to the current infection.

### 2.3. Imaging and Instrumental Examinations

**Figure 1:**
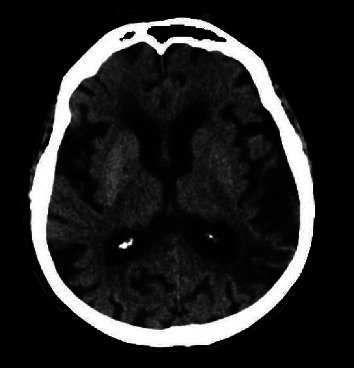
Patient's noncontrast brain computed tomography.

A noncontrast brain computed tomography revealed hyperdensity of the right basal nuclei, as depicted in Figure 1.

**Figure 2:**
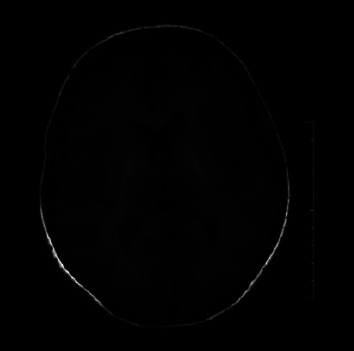
Patient's noncontrast brain magnetic resonance (on *T*1 weighted).

Afterwards, the patient underwent magnetic resonance that revealed hyperintensity on *T*1 in both the basal ganglia associated with areas of decreased signal intensity on *T*2 with pigments deposition (see Figure 2).

An electroencephalogram was also performed: it demonstrated nonspecific slow waves without spikes.

### 2.4. Therapeutic Approach

The patient received fluids, insulin, and symptomatic treatment to control choreiform and ballistic movements (clonazepam). *Staphylococcus aureus* was detected in the urine. Piperacillin-tazobactam therapy was administered.

Supplements with iron, folic acid, and B12 vitamins were administered due to deficiencies.

After two weeks, symptoms improved as well as the glucose control, and then she was discharged.

Oral antidiabetic dosage was improved, without insulin therapy because of sufficient insulin reserves. A close diabetes follow-up was organised. Nevertheless, the patients did not come to the following appointments.

## 3. Discussion

This case report of diabetic striatopathy (DS) is similar to others described [1, 3]. Now the challenge is the understanding of the pathogenic mechanisms of this condition.

Chorea is a hyperkinetic dyskinesia characterized as being involuntary, irregular, unpredictable, and small-amplitude. Ballismus is featured as being large-amplitude, arrhythmic, and more proximal. Chorea-ballismus can appear in the large majority of patients with DS [1].

Chorea-ballismus can also be observed in some ketotic diabetic patients, adolescents with newly diagnosed diabetes, and even children. In the elderly, other causes that could induce acute/subacute-onset choreiform movement must be considered: ischemic/hemorrhagic stroke, rheumatologic diseases, vascular malformations, electrolyte imbalance (hyponatremia/hypernatremia, hypercalcemia, and hypomagnesemia), autoimmune neurologic syndromes/paraneoplastic syndromes, tumors, carbon monoxide intoxication, hyperthyroidism, and hypoparathyroidism/hyperparathyroidism, demyelinating disease, psychostimulants (cocaine and amphetamines), infections (viral encephalitis, cryptococcal granuloma, mycoplasma, tuberculoma, neurosyphilis, HIV encephalitis, and prion diseases), and drugs (neuroleptics, lithium, antiepileptic drugs, oral contraceptives, estrogen replacement therapy, steroids, methotrexate, cyclosporine and fluoroquinolones) [1, 2].

For our patient, the interaction of multiple mechanisms might explain the case: a hyperglycemic state due to an incipient infection together with vascular insufficiency in a poor-controlled diabetic patient.

The precise pathophysiology of DS is not well understood. Biopsy studies suggest that the lesion is a vasculopathy with gliosis restricted to the striatum. Postmortem findings have been somewhat inconsistent but include reactive astrocytosis, patchy ischemic necrosis, petechial hemorrhage, vascular proliferation, and arteriolar changes similar to the diabetic proliferative retinopathy.

Our communication aims to remind clinicians to consider the possibility of diabetic striatopathy when poor-controlled diabetic patients have sudden-onset choreiform and ballistic movements. Moreover, this case suggests the possibility that oxidative and endoplasmic reticulum (ER) stress may be involved in this process. This is only speculative because we have not examined the molecules involved in these pathways and no in vitro-tests have been performed.

However, this is the very first time that these mechanisms have been mentioned as possible mediators in DS.

In fact, these actors (oxidative and ER stress) are very prominent in neurological disorders, in both ischemic and hemorrhagic stroke, and in diabetes itself. Chronic hyperglycemia-associated oxidative stress and low-grade inflammation are considered to play critical roles in the progression of diabetic complications [4, 5].

After intracerebral hemorrage, both oxidative and ER stress levels are upregulated and alleviating either ER or oxidative stress limits secondary neuronal damage. Using nicotinamide adenine dinucleotide phosphate oxidase inhibitors and nonspecific reactive oxygen species scavengers, oxidative stress is reduced, cerebral vascular function improves, and cerebral amyloid angiopathy-related microhemorrhages are reduced. Reactive astrogliosis and apoptosis have been shown to be reduced using antioxidant compounds [6].

DS is a very uncommon disease, but its knowledge should be mandatory for physicians, and its mechanisms have to be better investigated. This case report adds same knowledge about this rare condition together with previous works [1, 3].

To conclude, in the future, the detection of circulating markers of oxidative and ER stress should be given serious consideration in patients with DS, in order to eventually propose antioxidant therapy and compounds ameliorating the ER stress, as is in the case of diabetes [7].

Postmortem studies in the brain tissues of DS patients should be focused on the detection of oxidative and ER stress markers. The results will strengthen or discard our hypothesis.

## Figures and Tables

**Table 1:** Main blood laboratory data at admission and discharge.

Blood laboratory data	Admission (Day 1)	Discharge (Day 14)
Haemoglobin (g/dL)	9.5	9.8
Hematocrit (%)	30	30.6
MCV (fL)	71	86
WBC (10^3^/mm^3^)	6220	8610
PLT per (mm^3^)	429	322
Glucose (mg/dL)	356	90
Glycated Haemoglobin (%)	10	9.8
C-peptide (ng/mL)	4	4
Creatinine (mg/dL)	0.6	0.7
Sodium (mEq/L)	138	136
Potassium (mEq/L)	3.8	3.4
Calcium (mg/dL)	10	10
Magnesium (mEq/L)	1.48	1.48
C-reactive protein (mg/L)	64	4
Ammonium (*μ*g/dL)	20	20
Iron (mcg/dL)	40	Not tested
Ferritin (mcg/L)	400	Not tested
Folates (ng/mL)	2	Not tested
B12 vitamin (pg/ml)	100	Not tested
Thyroid-stimulating hormone (mU/L)	2	Not tested
Urine culture	Positive: *Staphylococcus aureus*	Negative
Blood culture	Negative	Negative

## Data Availability

The data used to support the findings of this study are included within the article.
